# Reliability and validity of the Readiness for Hospital Discharge Scale in patients with spinal cord injury

**DOI:** 10.4102/sajp.v76i1.1400

**Published:** 2020-05-07

**Authors:** Jacques S. De Lange, Jolandi Jacobs, Nadine Meiring, Boitumelo Moroane, Tinei Verster, Steve Olorunju, Mokgadi K. Mashola

**Affiliations:** 1 Department of Physiotherapy, Faculty of Health Sciences, University of Pretoria, Pretoria, South Africa; 2 Biostatistics Unit, South African Medical Research Council, Pretoria, South Africa

**Keywords:** psychometric, spinal cord injury, validity, reliability, readiness for discharge, rehabilitation

## Abstract

**Background:**

Measuring rehabilitation outcomes in patients with spinal cord injury (PWSCI) requires measurement tools that are valid and reliable and have been psychometrically tested in the population with spinal cord injury (SCI). The Readiness for Hospital Discharge Scale (RHDS) has been found to be reliable and valid in adult surgical patients, post-partum mothers, parents of hospitalised children and geriatrics. However, the psychometric properties have not yet been tested in the population with SCI, furthermore, in a South African context.

**Objectives:**

The purpose of this study was to psychometrically test the internal consistency and construct validity of the RHDS as a measure of discharge readiness in PWSCI prior to discharge from rehabilitation units in the Tshwane metropolitan area, South Africa.

**Method:**

A cross-sectional study that included 50 PWSCI who were in their last week of rehabilitation was conducted. The RHDS item and scale statistics were calculated by using descriptive statistics and the scale reliability was measured for internal consistency by using Cronbach’s alpha coefficients. To determine construct validity, convergent and divergent validities were measured by using the RHDS items’ correlation coefficient dimensions. All data were tested at the 0.05 level of significance by using Statistics and Data (STATA) statistical software, version 14.

**Results:**

Cronbach’s alpha of the RHDS was 0.904, indicating an excellent reliability coefficient. Convergent validity scores showed 81% correlation coefficients, although divergent validity scores showed 62% correlation coefficients.

**Conclusion:**

The RHDS is a valid and reliable measure of readiness for discharge in a South African sample of PWSCI and can be used in SCI rehabilitation.

**Clinical implications:**

Over and above using the RHDS to determine if PWSCI are ready for discharge in the clinical setting, the RHDS may also assist health care practitioners to assess the patient’s progress towards readiness and strategies for addressing shortcomings to meet short and long-term goals of the rehabilitation process.

## Introduction

Evaluation of patient’s readiness for hospital discharge (RHD) after spinal cord injury (SCI) requires measurement tools that are valid and reliable. Psychometric testing of outcome measures provides the potential user with information on the quality of the outcome measure, thus assisting the user on which outcome measure would be best to use (de Souza, Alexandre & Guirardello 2017). It is important to know outcome measures in detail, such as items, domains or subscales and measurement properties before using them to ensure quality of results. The quality of the information provided by outcome measures will then depend on the psychometric property of the tool (de Souza et al. 2017). Measuring reliability and validity of outcome measures is important to remove biases and errors that could lead to inaccurate results (Kottner et al. 2011).

Readiness for hospital discharge refers to a patient’s preparedness to be able to leave an acute care facility (Weiss & Piacentine 2006). There is a growing need to assess RHD to ensure patient safety and life satisfaction at home after discharge. The Readiness for Hospital Discharge Scale (RHDS) was developed to measure if patients perceived themselves ready to be discharged from hospital and manage their care needs at a home setting (Weiss & Piacentine 2006). The RHDS also measures variables related to discharge readiness by teams of nurses, clinical specialists, and managers in the fields of adult care, maternal–neonatal and paediatric care.

Four attributes of patient’s perceptions of RHD are identified as personal status, knowledge, coping ability and expected support and are referred to as subscales of the questionnaire. The 21 items on the RHDS are presented in the form of questions, and answers require circling a number on a scale from 0 to 10 (Weiss & Piacentine 2006). The raters answer by circling the number that best describes how they feel. The higher the score on each item reflects higher RHD and total scores are obtained by summing the numeric responses to each item, with the highest possible score being 210 (Online Appendix).

The RHDS is used for post-partum patients (Weiss, Ryan & Lokken 2006), adult medical surgical patients (Weiss et al. 2007) and parents of hospitalised children (Weiss & Piacentine 2006) in the USA. Coffey and McCarthy (2013) used the RHDS in the geriatric population in Ireland. To our knowledge, the RHDS has been used only once in South Africa, in the SCI population (Du Plessis et al. 2018), without its psychometric properties being tested in a South African context and population. Perceptions of the expected support of South African patients with SCI (PWSCI) once discharged were found to be high (Du Plessis et al. 2018), leading to higher perceptions of RHD. However, it must be noted that the PWSCI reported an underwhelming moderate overall RHD, a finding that has been attributed to a general regimen of discharging PWSCI before being ready for community reintegration (Du Plessis et al. 2018).

Spinal cord injury significantly influences an individual’s entire lifestyle and has the potential to negatively influence their physical and psychological aspects of health and quality of life (Middleton, Tran & Craig 2007). People with SCI usually require protracted rehabilitation with the aim to enhance their functional independence in both their physical and psychosocial skills, and to prepare the PWSCI for transition from hospital to community living (Whiteneck et al. 2011). An important aspect in the rehabilitation is to ensure that PWSCI are well prepared for the transition from institutionalised rehabilitation to community living (Mothabeng 2011). However, the concept of ‘discharge readiness’ presents a challenge and can be influenced by numerous variables which sometimes make it difficult to determine whether the PWSCI is ready for discharge or not (Mothabeng 2011). The timing of discharging a PWSCI from rehabilitation is influenced to a large extent by the severity of the SCI and the rehabilitation progress of the patient (Mothabeng 2011). However, anecdotal evidence suggests that in some instances, PWSCI are discharged before they are ready to be discharged from the hospital or rehabilitation setting. A major challenge in a South African context is that there are limited facilities for the rehabilitation of PWSCI, resulting in long backlogs for admission (Sokhela et al. 2013). This puts pressure on the facilities to discharge PWSCI after a pre-determined period, whether they are ready for discharge or not. If PWSCI are discharged before they are ready, there may be serious consequences.

Problems associated with ‘unready for discharge’ include the development of secondary health conditions such as pressure ulcers and respiratory complications (Mashola & Mothabeng 2019), which are responsible for mortality in PWSCI (Guilcher et al. 2013) and readmission back to a hospital or a rehabilitation setting (Mashola, Olorunju & Mothabeng 2019). This is supported by Conradsson et al. (2018), who further suggest that having systemic approaches to the management of PWSCI in South Africa would improve their functional outcomes and reduce the mortality rates. Length of stay at a rehabilitation facility is a component of the established ‘SCI system of care’, and knowing when to appropriately discharge PWSCI from the rehabilitation facility forms an integral part of a holistic management of PWSCI (Divanoglou et al. 2010). Although a very short length of stay may be associated with adverse long-term outcomes, a longer length of stay does not necessarily suggest better long-term outcomes (Divanoglou et al. 2010). It is important for rehabilitation practitioners to be able to determine whether a patient is ready for discharge or not, by using standardised objective measures, as supported by Mansfield et al. (2016). Rehabilitation practitioners (such as physiotherapists) use measurement instruments, in different contexts for different reasons, including description, prediction and outcome evaluation. An important consideration in measurement is that instruments must be standardised and objective and must satisfy psychometric criteria of validity and reliability (Mothabeng 2011).

The 21-item RHDS was found to be reliable with a Cronbach’s alpha of 0.90 in a post-partum population, 0.93 in adult medical surgical patients and 0.83 in parents of hospitalised children (Weiss & Piacentine 2006). Confirmatory factor analysis confirmed a four-structure factor that included personal status, knowledge, coping ability and expected support subscales. Weiss and Piacentine (2006) further confirmed the validity of the RHDS, with the contrast group comparisons supporting the construct validity and predictive validity assessment supporting the RHDS as a measure of the patient’s perceptions of RHD. Weiss and Piacentine (2006:14) suggested that the RHDS would be useful to evaluate RHD for a ‘broad range of patient types’.

Although used by Du Plessis et al. (2018), the RHDS has not yet been tested for reliability and validity in a SCI population, nor in South Africa, hence the need for our study. Our study evaluated the internal consistency and construct validity of the RHDS in PWSCI in South Africa. A reliable and valid tool to determine RHD in a SCI population will add to the holistic management of PWSCI in determining appropriate length of stay, to prevent early discharge from rehabilitation.

## Methods

A cross-sectional observational study was conducted to collect data at three public and two private SCI rehabilitation units in the Tshwane metropolitan area in the northern region of the Gauteng Province, South Africa, for this psychometric analysis. Patients with SCI who were older than 18 years and within 1 week of discharge from rehabilitation were invited through their treating physiotherapists to participate in our study.

Participants needed to be able to speak or understand any of the 11 South African national languages to be included in our study. The authors were able to speak English, Zulu, Afrikaans, Sepedi, Sesotho and Setswana.

A translator was available for participants who could not speak any of these languages. A non-probability, convenience sampling method was used and 50 consenting PWSCI were included in our study, irrespective of cause, type, level or classification of SCI. The American Spinal Injury Association (ASIA) Impairment Scale was used to classify the SCI as complete or incomplete. Complete SCI refers to an absence of all motor and sensory functions, including the sacral segments below the level of injury, and an SCI is incomplete if there is some preservation of motor or sensory functions below the level of injury (Roberts, Leonard & Cepela 2017).

## Data collection tools and procedure

A socio-demographic and injury profile data capture sheet was used to collect demographic data such as age, gender and injury profile. Data pertaining to RHD were collected by using the RHDS that took 10 min to complete. The pain and stress items are reversely scored. We conducted our study concurrently with the study by Du Plessis et al. (2018), as a two-phase study investigating RHD as part of a larger SCI rehabilitation outcomes project.

The two teams collected the RHDS data. Du Plessis et al. (2018) measured the level of readiness and the factors associated therewith and reported the perceived RHD between PWSCI and their treating physiotherapists. Our study psychometrically analysed the RHDS data, which is reported in this article. The treating physiotherapists informed the authors of PWSCI who were within 1 week of discharge and an appointment was made with the patients. The authors first explained our study to the potential participants, and if they met the inclusion criteria and consented to participate, demographic and RHDS data were collected from them.

## Statistical analysis

The RHDS data were captured onto a Microsoft Excel spreadsheet, and the psychometric analyses were computed for reliability and validity aspects by using version 14 of the STATA statistical software (StataCorp 2015). The RHDS item and scale statistics were calculated by using descriptive statistics, and we measured the scale reliability of the RHDS for internal consistency by using Cronbach’s alpha coefficients. Internal consistency reliability addresses the extent to which all items on an instrument measure the same variable (Brink, Van der Walt & Van Rensburg 2012) and should be > 0.70 for the instrument to be reliable (Horner & Larmer 2006).

Our study investigated the construct validity of the RHDS, to ensure that the instrument does not contain elements that capture unrelated content, and ensure that the instrument actually measures the construct it is intended to measure (Bolarinwa 2015). Convergent validity tests for correlations with other instruments intending to measure the same or similar concepts, and divergent validity tests for a lack of correlations with instruments that assess different concepts (Horner & Larmer 2006). Convergent validity was determined by the RHDS items having a correlation coefficient with a score of their own dimensions greater than 0.40 (Stewart, Hays & Ware 1988). The RHDS items having a correlation coefficient with a score of their own dimensions, greater than those computed with other scores, determined the divergent validity. All data were tested at the 0.05 level of significance.

### Ethical consideration

Ethical approval for our study was granted by the Research Ethics Committee of the Faculty of Health Sciences University of Pretoria (reference number: 412/2016). Written informed consent was given by all participants prior to commencement of the study.

## Results

### Demographic characteristics

Fifty PWSCI participated in our study. The 50 participants consisted of 30 males (60%) and 20 females (40%). Paraplegia was the most common type of SCI (70%, *n* = 35), with 60% (*n* = 30) of the injury being incomplete (Table 1).

**TABLE 1:** The demographic characteristics of patients with spinal cord injury (*n* = 50).

Demographic characteristics		Number	Percentage
Gender	Male	30	60
	Female	20	40
Age in years	18–29	12	24
	30–39	8	16
	40–49	14	28
	50–59	9	18
	>60	7	14
Discharge setting	Home	41	82
	Rehabilitation setting	8	16
	Other	1	2
Discharged residential area	Township	17	34
	Suburb	20	40
	Informal settlement	7	14
	Other	6	12
Who do you live with?	Own family	48	96
	Relatives	1	2
	Other	1	2
Is help needed at home?	No	17	34
	Yes	33	66
Is there help at home?	Not applicable	15	30
	No	1	2
	Yes	34	68
Type of SCI	Paraplegia	35	70
	Tetraplegia	15	30
Level of SCI	C1–C4	5	10
	C5–T1	15	30
	T2–T6	11	22
	T7–T12	9	18
	L1–L5	9	18
	S1–S5	1	2
Completeness of SCI	Complete	11	22
	Incomplete	30	60
	Don’t know	9	18

SCI, spinal cord injury.

### Psychometric analysis of the Readiness for Hospital Discharge Scale

The item descriptive characteristics of the RHDS are presented in Table 2.

**TABLE 2:** The Readiness for Hospital Discharge Scale item statistics.

Item	*n*	Mean	Standard deviation
Physical RHD	50	7.86	2.041
Pain†	50	2.46	2.697
Strength	50	6.96	1.979
Energy	50	7.14	2.109
Emotional RHD	50	8.48	2.367
Physical self-care	50	7.14	2.277
Stress†	50	2.46	2.644
Know about self-care	50	7.52	2.720
Know about medical needs	50	7.88	2.438
Know about problems	50	7.68	2.360
Know who to call if problems	50	7.28	2.857
Know restrictions	50	7.24	2.700
Know about follow-up	50	6.24	3.274
Know about resources	50	6.60	3.149
How well to handle demands	50	7.42	2.339
How well to perform self-care	50	7.70	2.053
How well to perform medical treatment	50	8.22	2.122
Emotional support at home	50	9.14	1.309
Help personal care at home	50	8.80	1.796
Help with household activities	50	8.26	2.230
Help with medical needs	50	8.30	2.013

RHD, readiness for hospital discharge.

† , Items are reversely scored.

#### Internal consistency reliability

The RHDS reliability characteristics are presented in Table 3, with the overall test scale evaluated through average inter-item correlation matrices, which ranged from 0.295 to 0.336. Cronbach’s alpha for the RHDS instrument was 0.904.

**TABLE 3:** Test scale for the Readiness for Hospital Discharge Scale.

Item	Item–test correlation	Item–retest correlation	Average inter-item correlation	Alpha
Physical RHD	0.6027	0.5469	0.3077	0.8989
Pain†	0.1587	0.0780	0.3364	0.9102
Strength	0.5843	0.5268	0.3089	0.8994
Energy	0.6074	0.5521	0.3074	0.8988
Emotional RHD	0.5812	0.5233	0.3091	0.8995
Physical self-care	0.6091	0.5539	0.3073	0.8987
Stress†	0.3008	0.2241	0.3272	0.9068
Know about self-care	0.6846	0.6375	0.3024	0.8966
Know about medical needs	0.6550	0.6046	0.3043	0.8974
Know about problems	0.6464	0.5950	0.3049	0.8977
Know who to call if problems	0.5942	0.5375	0.3083	0.8991
Know restrictions	0.6680	0.6190	0.3035	0.8971
Know about follow-up	0.5372	0.4754	0.3119	0.9007
Know about resources	0.5756	0.5171	0.3095	0.8996
How well to handle demands	0.4317	0.3620	0.3188	0.9035
How well to perform self-care	0.7003	0.6550	0.3014	0.8961
How well to perform medical treatment	0.5638	0.5043	0.3102	0.9000
Emotional support at home	0.6978	0.6523	0.3016	0.8962
Help personal care at home	0.7986	0.7660	0.2951	0.8933
Help with household activities	0.5401	0.4786	0.3118	0.9006
Help with medical needs	0.7397	0.6993	0.2989	0.8950
Test scale	-	-	0.3089	0.9037

RHD, readiness for hospital discharge.

† , Items are reversely scored.

#### Convergent and divergent validity

Table 4 shows the correlation matrix of the RHDS. The RHDS has 17 out of 21 items (81%) with a correlation coefficient with the score of their own dimensions greater than 0.40. This instrument as used by PWSCI therefore satisfies the criterion for item convergent validity (*r* ≥ 0.40) as established by Stewart et al. (1988). For divergent validity, the RHDS had 13 out of 21 items (61.9%) with a correlation coefficient with the score of their own dimensions, greater than those computed with other scores.

**TABLE 4:** Correlation matrix of the Readiness for Hospital Discharge Scale.

Item	Personal status	Knowledge	Coping ability	Support
Physical RHD	0.436	0.372	0.275	0.464
Pain†	−0.167	−0.004	−0.084	0.050
Strength	0.404	0.370	0.404	0.307
Energy	0.342	0.347	0.308	0.485
Emotional RHD	0.407	0.273	0.352	0.638
Physical self-care	0.548	0.376	0.435	0.527
Stress†	−0.217	−0.102	−0.072	−0.301
Know about self-care	0.433	0.665	0.517	0.502
Know about medical needs	0.406	0.643	0.430	0.471
Know about problems	0.317	0.669	0.449	0.352
Know who to call if problems	0.322	0.691	0.314	0.351
Know restrictions	0.314	0.637	0.399	0.503
Know about follow-up	0.301	0.541	0.484	0.308
Know about resources	0.232	0.547	0.549	0.378
How well handle demands	0.113	0.409	0.361	0.301
How well perform self-care	0.594	0.506	0.428	0.497
How well perform medical treatment	0.325	0.481	0.512	0.378
Emotional support at home	0.513	0.516	0.452	0.710
Help personal care at home	0.581	0.614	0.504	0.849
Help with household activities	0.486	0.243	0.289	0.556
Help with medical needs	0.439	0.529	0.504	0.693

RHD, readiness for hospital discharge.

† , Items are reversely scored.

## Discussion

Our study examined the validity and reliability of the RHDS as a measure of perception of RHD in a population of South African PWSCI. The RHDS was shown to be reliable in determining a patient’s readiness to be discharged from hospital in the PWSCI population, with a Cronbach’s alpha of 0.904. This result is regarded as an excellent reliability coefficient. Similar results were reported by Weiss and Piacentine (2006) as well as Coffey and McCarthy (2013), who found the RHDS to have a Cronbach’s alpha value of 0.93, 0.90, 0.83 and 0.73 in populations, including adult medical surgical patients, post-partum mothers, parents of hospitalised children and geriatrics, respectively. The high internal consistency of the RHDS indicates that the scale’s items are homogenous; in other words, the items are all measuring the same attributes in this population of PWSCI. The item convergent and divergent validities for the RHDS were established, suggesting that the RHDS is a true measure of perception of RHD for PWSCI.

The patient’s perception of RHD is an important part of rehabilitation care and investigating it in depth would add vital knowledge to the current ‘SCI system of care’ when determining length of hospital stay. The discharge period has been correctly identified as a time to evaluate the hospital-based care and assess potential risks for future complications (Weiss & Piacentine 2006), and using the RHDS will be a beneficial tool to evaluate these. There is a current need to improve the outcome of SCI in South Africa, including the need to monitor the timing of essential processes of care in relation to secondary health complications and long-term outcomes (Conradsson et al. 2018). Using the RHDS will be useful in clinical practice to screen for discharge readiness to prevent unnecessary prolonged hospital stays (and ultimately reduce overall cost of stay), as well as to target intervention plans necessary to reduce coping difficulty post-discharge. Furthermore, the RHDS can be useful for research purposes, when used in outcome studies evaluating the transition to post-discharge care as suggested by Weiss and Piacentine (2006).

### Strengths and limitations

Our study sought to determine the psychometric properties of the RHDS in the South African context of PWSCI. Our study contributes to literature of RHD and establishing. Our study has demonstrated satisfactory reliability and validity of the RHDS in the PWSCI population, similar to the findings by Weiss and Piacentine (2006) in various other populations. Although our study was able to include all PWSCI who were discharged at the selected settings, a larger sample size would have been preferable. These psychometric data are based on data from a cross-sectional study and as such, time-dependent aspects such as predictive validity and test–retest reliability could not be established. Furthermore, inter-rater reliability could not be established, as our study did not have more than one assessor rating one patient (Horner & Larmer 2006).

### Practical implication and recommendations

Results of our study provide evidence that the 21-item RHDS is a valid and reliable questionnaire to use in the population with SCI. Our study provides a platform for physiotherapists and healthcare practitioners in a hospital or rehabilitation setting with an evidence-based, objective method to determine whether their patients with SCI are ready for discharge. The authors recommend that further psychometric testing be performed to establish sensitivity and responsiveness of the RHDS to complete the assessment of the psychometric properties of the scale. We recommend that the RHDS be used in clinical practice to determine RHD of PWSCI in a South African context. For future studies, the authors recommend implementing a similar study with the same population over a longer period of time, which will allow a larger sample size, producing more accurate results as well as implementing the RHDS in planning readiness for discharge in different patient populations.

## Conclusion

The RHDS is a reliable and valid tool to measure the extent to which PWSCI measure their perception of being ready for hospital discharge. The RHDS was found to be psychometrically sound with excellent reliability coefficients and is therefore suitable to be used among South African PWSCI.
